# Advice from a Medical Expert through the Internet on Queries about AIDS and Hepatitis: Analysis of a Pilot Experiment

**DOI:** 10.1371/journal.pmed.0030256

**Published:** 2006-07-04

**Authors:** Javier Marco, Raquel Barba, Juan E Losa, Carlos Martínez de la Serna, María Sainz, Isabel Fernández Lantigua, Jose Luis de la Serna

**Affiliations:** **1**Hospital Clínico San Carlos, Madrid, Spain; **2**Fundación Hospital Alcorcón, Alcorcón, Madrid, Spain; **3***El Mundo,* Madrid, Spain; Pew Internet and American Life ProjectUnited States of America

## Abstract

**Background:**

Advice from a medical expert on concerns and queries expressed anonymously through the Internet by patients and later posted on the Web, offers a new type of patient–doctor relationship. The aim of the current study was to perform a descriptive analysis of questions about AIDS and hepatitis made to an infectious disease expert and sent through the Internet to a consumer-oriented Web site in the Spanish language.

**Methods and Findings:**

Questions were e-mailed and the questions and answers were posted anonymously in the “expert-advice” section of a Web site focused on AIDS and hepatitis. We performed a descriptive study and a temporal analysis of the questions received in the first 12 months after the launch of the site. A total of 899 questions were received from December 2003 to November 2004, with a marked linear growth pattern. Questions originated in Spain in 68% of cases and 32% came from Latin America (the Caribbean, Central America, and South America). Eighty percent of the senders were male. Most of the questions concerned HIV infection (79%) with many fewer on hepatitis (17%) **.** The highest numbers of questions were submitted just after the weekend (37% of questions were made on Mondays and Tuesdays). Risk factors for contracting HIV infection were the most frequent concern (69%), followed by the window period for detection (12.6%), laboratory results (5.9%), symptoms (4.7%), diagnosis (2.7%), and treatment (2.2%).

**Conclusions:**

Our results confirm a great demand for this type of “ask-the-expert” Internet service, at least for AIDS and hepatitis. Factors such as anonymity, free access, and immediate answers have been key factors in its success.

## Editors' Summary

**Background.:**

Although substantial progress has been made in the fight against HIV/AIDS, in terms of developing new treatments and understanding factors that cause the disease to worsen, putting this knowledge into practice can be difficult. Two main barriers exist that can prevent individuals seeking information or treatment. The first is the considerable social stigma still associated with HIV; the second is the poverty of the developing countries—such as those in Latin America—where the disease has reached pandemic proportions. In addition, the disease, which used to be spread mainly through the sharing of injecting drug needles or through sex between men, has now entered the general population. When healthcare services are limited, people are often unable to seek information about HIV, and even when services do exist, the cost of accessing them can be too high. The same is true for other diseases such as hepatitis infection, which often co-exists with HIV. The Internet has the potential to go some way to filling this health information gap. And, many patients seek information on the Internet before consulting their doctor.

**Why Was This Study Done?:**

In 2003, the Madrid-based newspaper *El Mundo* launched an HIV and hepatitis information resource situated in the health section of its existing Web site. One aspect of this resource was an “ask-the-expert” section, in which readers could anonymously e-mail questions about HIV and hepatitis that would be answered by an infectious disease expert. These ranged from how the diseases can be transmitted and who is most at risk, to what to do if an individual thinks they might have the disease. There seems to be a clear need for this Spanish-language service; in Latin America, 2.1 million people are infected with HIV, with 230,000 new cases in 2005. In the Caribbean, AIDS is the leading cause of death in people aged 15–44 years. In Spain, 71,000 people were infected with HIV in 2005. Although the Internet contains a vast store of health information, and many aspects of patient–doctor interactions have been made electronic, little is known about what format is ideal. The researchers, who included employees of the newspaper, decided to investigate the effectiveness of the question–answer format used by *El Mundo*.

**What Did the Researchers Do and Find?:**

In the first 12 months after the service was launched, the researchers recorded several details: what day of the week questions were sent, what the questions were about, and whether they were sent by the person needing the information or by a family member or friend. They also noted demographic information, such as the age, sex, and country of origin of the person e-mailing the question.

Of 899 questions sent to the Web site between December 2003 and November 2004, most (80%) were sent by males. Most questions came from Spain, followed by Latin America, and most questions were sent on Mondays and Tuesdays. Some e-mails were from people who felt they had been waiting too long for an answer to their first e-mail—despite the mean time for answering a question being fewer than seven days. Messages of support for the Web site rose during the year from 2% to 22%.

**What Do These Findings Mean?:**

The messages of support and encouragement sent in by users indicated that the service was well-received and useful. Most of the questions were about HIV rather than about hepatitis, which the researchers say could represent the more prominent media coverage of HIV. However, despite the disease's high profile, the questions about HIV were very basic. It could also mean that people hold a false impression that hepatitis is a less serious illness or that they have more information about it than about HIV.

Since most questions were sent in at the start of the week, the researchers believe that many individuals wrote in after engaging in potentially risky sexual behaviour over the weekend.

The researchers also found that existing information on the Web site already answered many of the new questions, indicating that people prefer a question-and-answer model over ready-prepared information. The anonymity, free access, and immediacy of the Internet-based service suggest this could be a model for providing other types of health information.

The findings also suggest that such a service can highlight the needs and concerns of specific populations and can help health planners and policymakers respond to those needs in their countries.

**Additional Information.:**

Please access these Web sites via the online version of this summary at http://dx.doi.org/10.1371/journal.pmed.0030256.

• The AIDSinfo Web site from the US Department of Health and Human Services provides information on all aspects of HIV/AIDS treatment and prevention and has sections specially written for patients and the general public

• AVERT, an international AIDS charity, has a section on HIV in Latin America that includes details of transmission, infection rates, and treatment

## Abstract 3

Marco and colleagues analyzed questions sent by the public to a Spanish language "ask-the-expert" Internet site, and found that 70% of queries were about risk factors for acquiring HIV.

## Introduction

AIDS can be considered the first media disease. Since its discovery in 1981, HIV infection has had a constant presence in the public eye. Its obscure origin and lethality, the fact that celebrities were affected by the disease, and its initial relationship with homosexuality, drug abuse, or racial origin made HIV an immediate public concern and subject in the media. Today things have changed: AIDS is a treatable disease and an example of how an enormous research effort can bring about dramatic results in the knowledge and management of a new medical problem. The AIDS epidemic has changed dramatically since its beginning. In Western Europe and North America it has slowed its progress, while other parts of the world such as Africa and Asia are suffering a devastating pandemic. The latest survey shows a total of 720,000 HIV-infected patients in Western Europe with an estimated 22,000 new cases in 2005 [ 1]. Although sex between men and in a few countries drug use through injection remain important routes for HIV transmission, increasing numbers of people are being infected through unprotected heterosexual intercourse. In Spain the number of infected individuals is 71,039, with 1,712 new cases in 2005 [ 2]. The route of infection has changed in developed countries from most transmission being between males through sexual intercourse and intravenous drug use in the last decade to the current predominance of heterosexual transmission and rise in the number of infected women. Estimates from Latin America (the Caribbean, Central America, and South America) show a total of 2.1 million HIV-infected patients with 230,000 new cases in 2005. The AIDS epidemic claimed an estimated 24,000 lives in the Caribbean in 2005, making it the leading cause of death among adults aged 15–44 years. The Caribbean's status as the second-most affected region in the world masks substantial differences in the extent and intensity of its epidemics, from an estimated 3% in Haiti to less than 0.2% in Cuba.

The number of people living with HIV in Latin America has risen to an estimated 1.8 million. In 2005, approximately 66,000 people died of AIDS, and 200,000 were newly infected. Primarily due to their large populations, the South American countries of Argentina, Brazil, and Colombia are home to the biggest epidemics in this region. Brazil alone accounts for more than one-third of the estimated 1.8 million people living with HIV in Latin America. Viral hepatitis C (VHC) frequently coexists with HIV infection. With the progress made on antiretroviral treatment, hepatic disease has become an important aspect in the management of HIV patients: use of antiretroviral drugs can be limited by liver toxicity, and end-stage hepatic disease is sometimes the main problem of co-infected subjects [ 3].

The Internet revolution has drastically changed aspects of daily life [ 4]. Factors such as its ubiquity and immediacy have opened a broad spectrum of new ways of interaction between individuals. Health management and services are fields where barriers and taboos have been broken earlier than in other activities: in a very short time patients and doctors have modified their ways, having been pushed by this electronic revolution [ 5– 7]. Today the Web is mainly used as an information and communication channel. Few studies have been done to evaluate which is the best format [ 7– 9] to improve and build new types of relationships between patients and doctors with this medium [ 10]. Recent data from the Health Information National Trends Survey in the United States portray an important change in the ways in which patients consume health and medical information, with more patients looking for information online before talking with their physicians [ 11]. The Internet provides access to information about the disease process in HIV/AIDS, clinical trials, medical treatments, alternative therapies, legal advice, social support, and access to services (e.g., chats with other patients, newsletters, glossaries, or directories of AIDS service organizations) [ 12]. On a smaller scale, similar information is available for viral hepatitis [ 13].

A considerable amount of research has been done on Internet use by HIV-infected patients. The existence of a digital divide has been a major concern in most of these studies. While people with a higher level of education and income have fairly good access to Internet resources and are able to understand the information given, a considerable number of people are still on the blind side of this revolution [ 14– 16]. Access to Internet resources has been shown to have an important impact on HIV/AIDS. Individuals with access to the Web, particularly to health information, are among those with better resources and are healthier persons living with the disease. Health-related Internet use is associated with a broader spectrum of health behaviors including HIV treatment adherence and active coping strategies with potential health benefits [ 14].

Looking at the wide variety of initiatives now under way, giving expert medical advice about questions and concerns expressed anonymously by patients through the Internet and made public on the Web offers clear advantages. If we place this tool in a channel of general information and make it available 24 hours a day, seven days a week, we end up with a tremendously active and constantly changing interaction. AIDS and hepatitis C have their own unique attributes on a site with these characteristics. Both are diseases with great impact on the media. They also need chronic and complex medical management, and toxic side effects are common. The general population is deeply concerned about them because of their contagiousness rather than because of their widespread existence [ 13]. With the changing pattern of the AIDS epidemic from one with specific risk behaviours to more general heterosexual transmission, the disease has entered into everybody's daily life.

In December 2003 the Spanish newspaper *El Mundo,* through its online edition ( http://www.elmundo.es) launched a new Internet site centred on different aspects of HIV infection and viral hepatitis, including an “ask-the-expert” section. According to the official Spanish agency that registers Internet traffic (Oficina de Justificación de la Difusion), in October 2005 http://www.elmundo.es was the largest Internet newspaper in the Spanish language with 7,252,353 unique users monthly, and *elmundosalud.com* (the health section of this Web site) received six million accesses with 830,000 unique users during this same month. An HIV/AIDS and VHC consumer-oriented Internet resource that is directly accessible through a news media outlet and which has great impact on the targeted population offers a unique opportunity for health education and is an excellent tool for identifying patient needs and the information demands of the general public. The present paper analyses an Internet service of “ask the expert” on advice about HIV/AIDS and hepatitis of the aforementioned characteristics in a Spanish-speaking population.

### Aim of the Study

The aim of this study was to perform a descriptive analysis of questions about AIDS and hepatitis made to an infectious disease expert sent through the Internet to a consumer-oriented website in the Spanish language. Answers to the questions had been posted on the Web site along with the questions (which were presented anonymously). We evaluated complementary aspects such as demographic characteristics of our users, type of questions, age of questioner, and the evolution of the service in its first 12 months of life.

## Material and Methods

**Figure 1:**
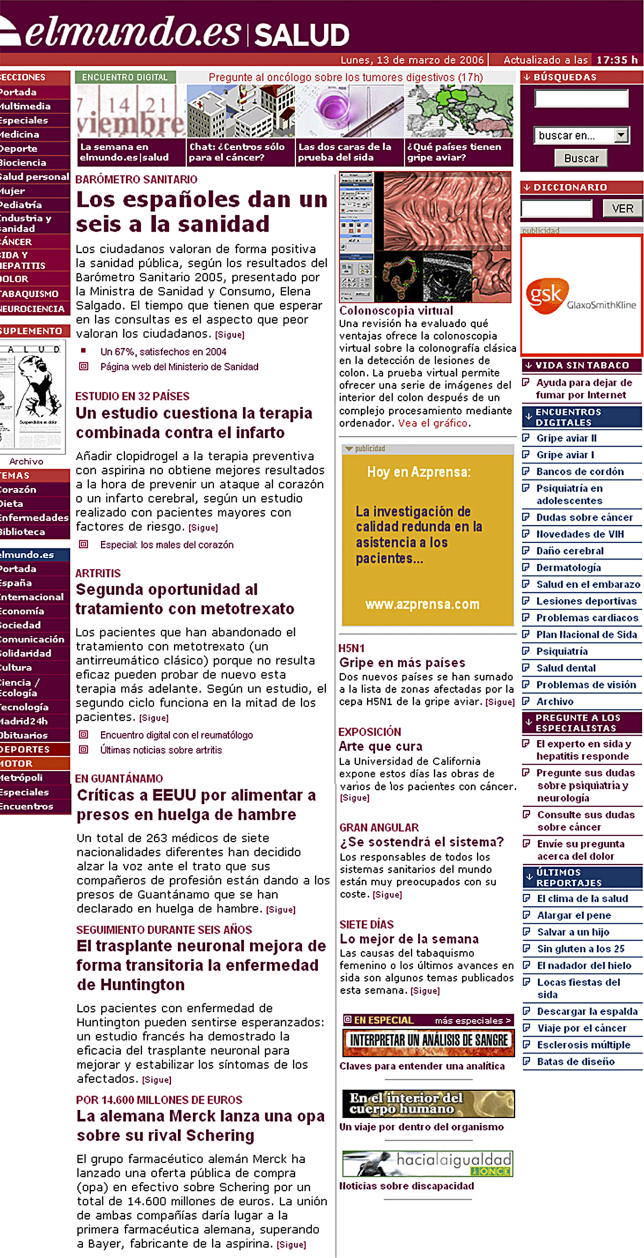
Homepage of *elmundosalud.com*

**Figure 2:**
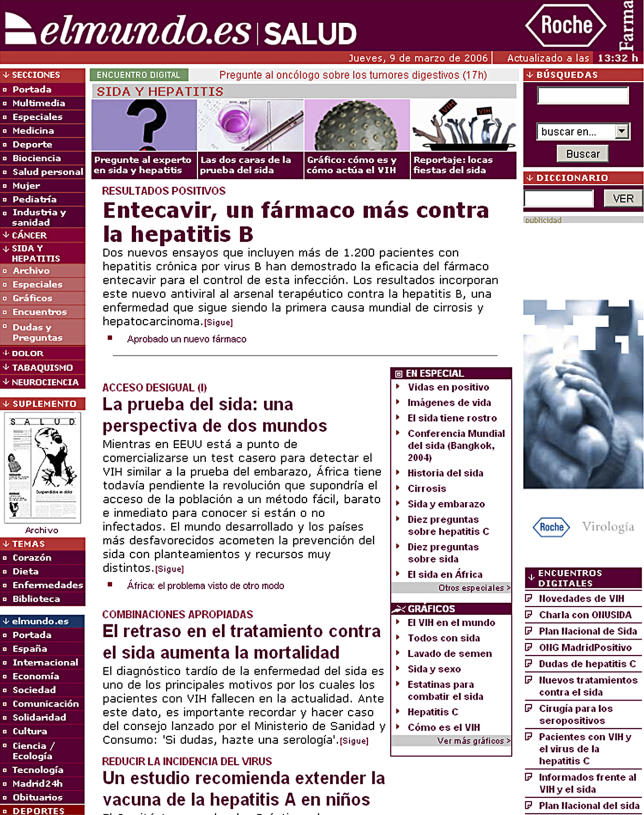
Home Page of the AIDS/Hepatitis Section

**Figure 3:**
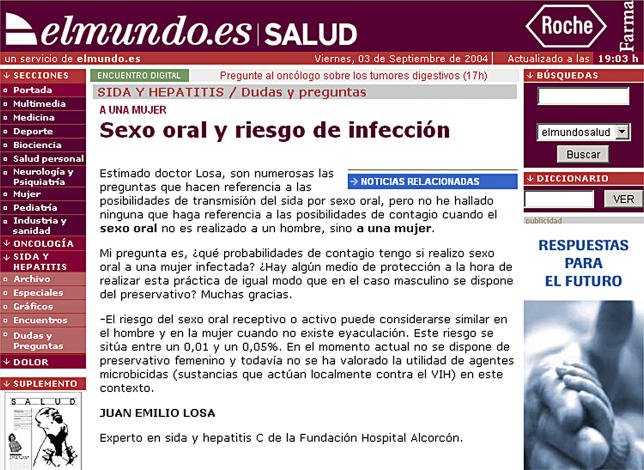
Example of an Answered Question on the Web *Translation of Question and Answer:* Question on oral sex and the risk of infection: Dear Doctor Losa, although there are numerous questions addressing the risk of AIDS transmission through oral sex, I have not been able to find any referring to this risk when oral sex is given by a man to a woman. My question is: what are my chances of contracting AIDS if I give oral sex to an infected woman? Is there a way to perform this practice safely similar to the condom used by men? Thank you. Answer to the female questioner: The risk for either receptive or active oral sex is very similar in men and women. This risk ranges between 0.01% and 0.05%. No feminine condoms are available at the present moment, and the efficacy of microbicide agents (compounds active locally against HIV) has not been evaluated in this setting. JUAN EMILIO LOSA, expert on AIDS and hepatitis, Alcorcón Hospital Foundation

The AIDS/hepatitis Web site is sponsored by the pharmaceutical industry (Roche Laboratories, Nutley, New Jersey). The company pays a fixed yearly amount to the newspaper for the presence of a publicity banner on the Web site. There is no other contractual obligation between the partners, and by written accord there is no interference with content nor is the Web site subject to any kind of control by the sponsor. The medical expert has no relationship with the sponsoring company. Access to the site is made through *elmundosalud.com* ( Figure 1), ( http://www.elmundo.es/elmundosalud), which is the home page of the more general health site host, and which includes specific pages about tobacco, cancer, and pain. There is an active link on most of the pages that connect to the AIDS and hepatitis pages ( Figure 2) ( http://www.elmundo.es/elmundosalud/hepatitissida/index.html). The hepatitis and AIDS pages are divided into sections on news, special information, chats, interactive graphics, and “expert advice” ( Figure 3) ( http://www.elmundo.es/elmundosalud/hepatitissida/dudasypreguntas.html). Figures 1– 3 show screen shots of these pages. Questions e-mailed by users are answered by an infectious disease expert with special interest in AIDS and viral hepatitis (JEL, PhD, MD). Queries were sent to him on a weekday daily basis (Monday through Friday) and the questions with their answers were posted anonymously (as soon as they were returned by the doctor) by Internet editors in the “expert-advice” section. For questions very similar to those already published, some standardized answers are e-mailed directly to the subject. The sender only receives an e-mailed answer if the question will not be posted anew on the Web. Published answers generally carry links to other interactive material, to related answers, or to recommended sites offering additional information. There is also a search engine to look for previous questions and their answers. Data identifying the sender is never disclosed. Editorial changes to the original text of the questions are minimal to preserve the layman's language as much as possible.

Every question received in the first 12 months was analyzed for the following variables: date (day of the week and month) on which the e-mail was sent, the presence in the text of a request for an urgent or speedy answer, subject of the question (whether it was about AIDS or hepatitis C, risk factors, route of infection, treatment, or prevention), and if the solicited information was for the sender, a friend, or a family member. We also took into account if it was a first or a follow-up question, and if there were present in the text support or encouragement for the Web site. We also registered demographic characteristics of the senders (age, sex, country of origin, and educational level) when it was possible to obtain this information from the text of the e-mail. The evolution in the number and type of queries for the successive months was also analysed. We performed a descriptive study and a temporal analysis of the questions. Bivariate relationships were determined using the chi-square test for categorical variables and *t-*test for continuous variables. All reported *p*-values are two-sided. *p*-Values of 0.05 or less were considered statistically significant. All statistical analyses were performed using SPSS 12.0.

## Results

A total of 899 questions were received from December 2003 until November 2004 with a marked linear growth pattern ( Figure 1). 703 of the questions were made by males (80.4%), with a mean age of 30.2 years (range 14–65) among the 66 e-mails that included this information. The country of origin was identified in 36.4% of cases: 66.4% came from Spain, 31.5% from Latin American countries, and 2.1% from other European nations. Mean age was significantly different depending on the type of question, country of origin, and gender of the person asking. People from Latin America, those interested in HIV, and males were younger ( Table 1). No significant difference between countries was found in the gender of the person sending the question: 26.7% of Latin Americans were female versus 19.5% in the Spanish population ( *p* = 0.2). Workdays had the heavier traffic, especially Mondays and Tuesdays (18.5% each). On weekends, this number was cut by half (9.9% on Saturdays and 8.5% on Sundays).

**Table 1:**
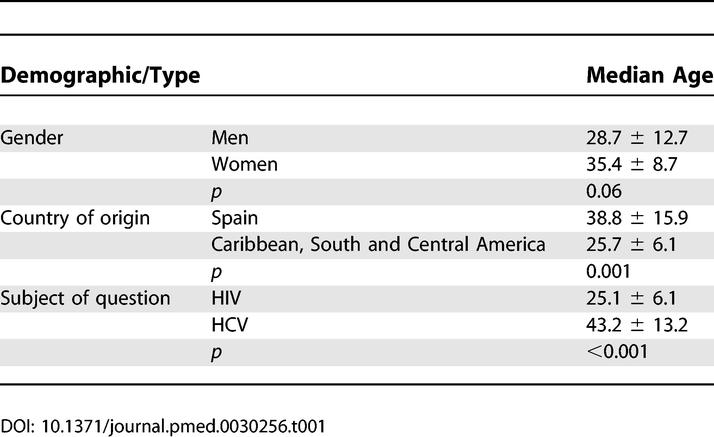
Median Age, Related to Demographic Characteristics and Topic of Question

**Figure 4:**
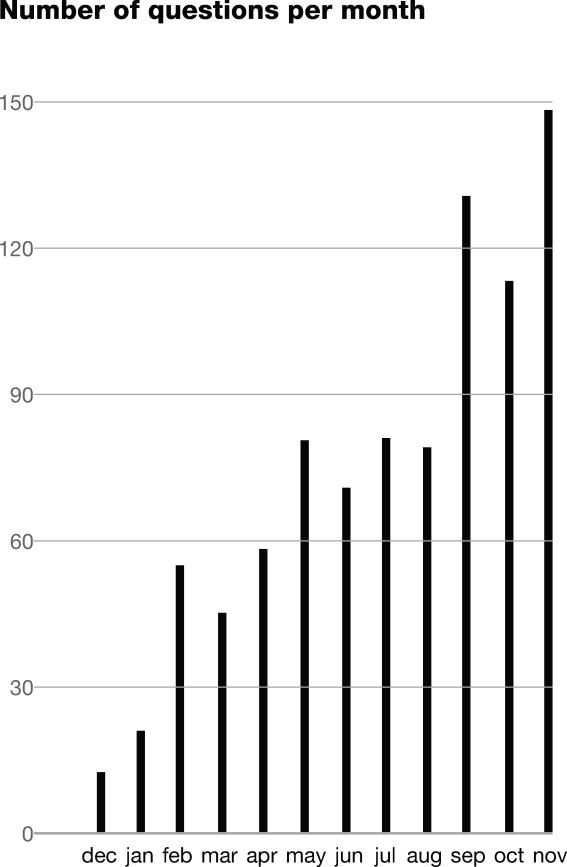
Number of Questions per Month

131 users (14.6%) sent more than one question, and this was a growing trend over time. Sometimes they wrote to enquire about their previously asked question (53%), other times to include new information (30.5%), and only 2.3% of the time with a new enquiry. Of the 899 questions, 48 (5.3%) considered their enquiry urgent and asked for an immediate reply. In general, the questions concerned the sender (only 7% were in reference to a friend or family member). The number of messages containing some sort of encouragement or support for the service was 204 (22.7%), with clear linear growth over time from 2% in January 2004 to 22% in November 2004 ( Figure 4). HIV infection was the exclusive or the main subject for most of the questions (82.7%). The rest were about hepatitis A, B, C, and other infections or different hepatitis types ( Table 2). One in three questions about risk factors for HIV infection was about oral sex (34%), 26.7% were about risk of infection through other routes such as saliva or skin contact, 12.3% were about unprotected sex, whereas 13.5% of users asked about the risk of contracting the disease after sex with protection. Most of the concerns about HIV were about the possibilities of infection for different risk behaviours (69%). Other questions concerned the window period (12.6%), laboratory results (6%), or disease symptoms (4.7%). Questions about HCV infection came mainly from Spain (80%). Of the 153 questions about HVC, 19% asked about the risk of infecting others, 15% requested advice about healthy living patterns, 13.1% about treatment efficacy, and 7.2% about routes of infection.

**Table 2:**
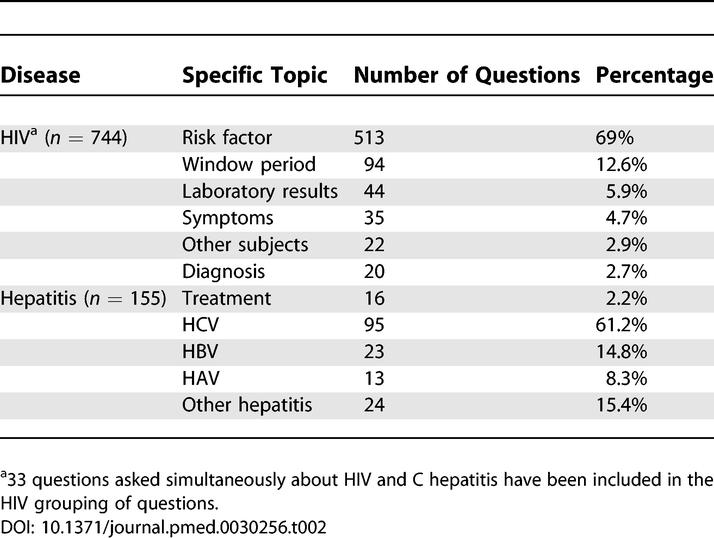
Types of Questions

## Discussion

Our results confirm a great demand for this type of “ask-the-expert” service through the Internet, at least for HIV and HCV infections. The use of this service shows in our experience a growth pattern typical of demand niches which are not covered by other types of services [ 17]. The mean age and predominance of the male sex is in accordance with other studies made in different countries [ 18]. The 14-year difference in median age between Spanish and Latin American users is surprising. As our method did not allow for registering this variable in a consistent way, it could be that only younger users felt it important to specify their age. Users seeking advice about HIV/AIDS were younger than those asking about hepatitis, although no significant age difference exists between these patients. This could be explained by a high number of doubts arising from young people after some sort of risky sexual relationship.

For a Spain-based Web site, the high number of users from Latin America is surprising and reflects the enormous reach of the Internet. The reason is probably multiple: information is more limited and access to social and health services is more restricted in Latin America than in Europe. Also, looking at the few questions originating in other European countries, the sharing of a common language (Spanish in this case) is another reason for the high usage in Latin America. Messages supporting the Web site were attached to questions, reflecting the constant growth of the site. Many of the messages were critical of the absence of similar initiatives or they praised different aspects our Web site, such as its seriousness, free access, quality, or the scientific content of the answers.

Most of the questions were directly related to HIV infection, with hepatitis clearly behind in interest. The high media exposure of AIDS compared with hepatitis is probably the main reason for this imbalance. Also, higher levels of information about hepatitis or the wrong impression that it is less serious than HIV could be behind these differences. The gap in the number of questions for the two diseases is more marked in Latin America where AIDS was clearly the main concern. The highest numbers of queries are sent just after the weekend, a fact most probably related with an increase in risky sexual practices during the weekend. The types of questions received suggest a low information level about AIDS among our users. Although one would think that with the high level of media exposure of AIDS/HIV, people using the Internet would have knowledge about the disease, but many of the questions addressed were very basic.

A considerable number of graphics and special documents were available on our Web site, illustrating and discussing many aspects present in the written questions. However, it seems that users prefer the question–answer model rather than ready-made information. It could be that a different profile exists for users on the Web who are not asking for direct advice. This latter group would have a higher level of education and be more knowledgeable about the disease and thus more capable of searching for and finding information directly.

Concerning hepatitis, in most of the cases it was the patient who was consulting about the risk of infecting household contacts. Most of the questions about hepatitis came from Spain, a fact that can be attributed to a lower level of awareness of this disease in Latin America. Compared with AIDS, we registered a higher percentage of questions about healthy living, treatment efficacy, and toxic effects for the different types of hepatitis. The level of information was higher than for HIV infection, especially in the questions coming from Latin America.

A considerable number of repeated questions were follow-ups of previous e-mail by the same sender. The mean time elapsed between question reception and answer posting was generally fewer than seven days, but it seems that this delay was considered excessive by some of our users. The Internet is perceived by users as a speedy, almost instantaneous means of communication, and an effort should be made to shorten these time lapses when interacting with them [ 5, 6]. After some months of the “ask-the-expert” service, we started receiving questions similar to some already posted. We opted for e-mailing personal replies to these people to avoid a reiterative section. Our e-mailed replies contained different types of standardised text as well as links to pertinent previous answers posted in the section. The main limitation of our study is the absence of some important information such as educational level or socioeconomic status to better describe our users.

Basic data such as age and country of origin was missing in some of the queries. We consciously decided not to use a proper questionnaire, in favour of confidentiality, easy access, and swift communication of standard e-mail. Although improbable, the profile of our users could be somewhat different from the one we describe. Also absent from our study are other important data such as expectations of the users, how the information given was used, and how the community as a whole might be using the information on the Web site. All this data was unfortunately out of our initial scope and should be obtained by specific questionnaires different from the simple circuit of e-mail and posting of answers that we used.

Looking at our results, it seems obvious that there is a great demand for this type of consultation service in the general population. In our opinion, factors such as anonymity, no fee charged, and immediacy of the answers are important keys for success. Perhaps one of the most interesting results of our study is that a general media information site, such as a newspaper on the Internet, turns out to be a good vehicle for delivering health information and advice. The media site delivered high Internet traffic, with a mean of four million accesses and 400,000 single users per month.

Sponsorship by the pharmaceutical industry can be a good working model if standards for independence are strictly followed. Users are sensitive to conflicts of interest in the healthcare industry. The Internet seems to be a practical source for a second opinion: it reduces cost, there is no waiting list, and the quality of the answer is guaranteed by a certified expert in the field [ 19] chosen by a well-known sponsor, such as the newspaper in this case. This experiment with two infectious entities, AIDS and hepatitis, both having an important impact on public health, revealed the Internet as an especially useful tool for disease awareness and prevention as well as for health education of patients and the public. Finally, this particular type of Internet health service offers the possibility of easy identification of patient needs and concerns in the general population. This knowledge can be important to health planners wanting to improve health policies and interventions.

### Conclusions

An “ask-the-expert” Internet resource for AIDS and hepatitis can be a good model for giving valid, widely sought information to patients in an anonymous, convenient, and timely way, and apparently it is in great demand. Its placement as a specialised site inside a health section that is part of a general information and news Web site appears to be a good model for this type of service, offering great accessibility to and exposure of the information. In view of its high demand and the increasing number of supportive messages received, our experience has been tremendously satisfactory. After two years, the Web site is well-established and its traffic continues to grow. Future investigations should employ a simple Web-based anonymous questionnaire including some questions about how the information given is used. This data will give a more comprehensive description of users and could be a very useful tool for tailoring content to their needs.

## Supporting Information

Alternative Language Abstract S1 Translation of the abstract into Spanish by Javier Marco (21 KB DOC)

Alternative Language Abstract S2 Translation of the abstract into French by Irene Marco (21 KB DOC)
