# Supplementary material for: Advice from a Medical Expert through the Internet on Queries about AIDS and Hepatitis: Analysis of a Pilot Experiment
Source: PLoS Med. 2006 Jul 4;3(7):e256. doi: 10.1371/journal.pmed.0030256 (PMC1483911; doi:10.1371/journal.pmed.0030256)
Supplement: Supplementary file 1 [file pmed.0030256.sd001.doc]

**Supporting information files**

**Translation of the abstract to Spanish by Javier Marco**

**RESÚMEN:**

**Introducción**: la publicación online de dudas y preguntas relacionadas con el SIDA y la Hepatitis enviadas de forma anónima mediante Internet y contestadas por un médico experto en el tema, supone un nuevo modelo de relación entre médico y paciente. El objetivo de este estudio es realizar un análisis descriptivo de las preguntas enviadas por Internet a un médico experto en enfermedades infecciosas sobre SIDA y Hepatitis localizado en un web en español dirigido al público general.

**Método y Resultados**: las preguntas se envían a través del correo electrónico ordinario y las respuestas contestadas se publican online en la sección “Dudas y Preguntas” de un web monográfico sobre SIDA y Hepatitis. Realizamos un estudio descriptivo y un análisis temporal de las preguntas recibidas durante los primeros doce meses de funcionamiento. Entre diciembre de 2003 y noviembre de 2004 se recibieron un total de 899 preguntas, con un marcado crecimiento lineal. Los correos procedían de España en el 68% de los casos, el 32% se enviaron desde Latinoamérica (Caribe, América Central y del Sur) y el 80% de los usuarios fueron varones. La mayoría de las dudas (79%) se referían a la infección VIH con la Hepatitis muy por detrás (17%). El mayor número de mensajes se recibía justo después del fin de semana (un 37% de las preguntas los lunes y martes). La preocupación más frecuente se refería a dudas sobre los factores de riesgo de contraer el VIH (69%9 seguido del período ventana (12,6%), resultados de análisis (5.9%), síntomas (4,7%), diagnóstico (2,7%) y tratamiento (2,2%).

**Conclusión**: Nuestros resultados confirman la elevada demanda de este tipo de servicio de internet de “consulte con el experto”, al menos en temas de SIDA y Hepatitis. El carácter anónimo, el acceso libre y la imediatez del servicio son factores claves que contribuyen al éxito de este tipo de experiencias.

**Conflicto de intereses: Ninguno. La web esta esponsorizada por la compañía farmacéutica Roche.**
