# Supplementary material for: Advice from a Medical Expert through the Internet on Queries about AIDS and Hepatitis: Analysis of a Pilot Experiment
Source: PLoS Med. 2006 Jul 4;3(7):e256. doi: 10.1371/journal.pmed.0030256 (PMC1483911; doi:10.1371/journal.pmed.0030256)
Supplement: Supplementary file 2 [file pmed.0030256.sd002.doc]

**Supporting information files**

**Translation of the abstract to French by Irene Marco**

**RESUME:**

**Introduction** : La publication en ligne de questions sur les thèmes du SIDA et de l’Hépatite posées par des navigateurs anonymes et des réponses apportées par un médecin spécialiste suppose un nouveau modèle de relation entre le médecin et le patient. Le but de cette recherche est de présenter une analyse descriptive des questions publiées dans une web espagnole qui se dirige au public général.

**Méthode et résultats:**

Las questions envoyées par les usuaires par courrier électronique ainsi que les réponses des médecins sont publiées dans la section “questions et doutes” d’une web monographique concernant le SIDA et l’Hépatite. Nous avons fait une recherche descriptive et une analyse temporelle des questions reçues pendant les douze premiers mois de fonctionnement. Les questions reçues entre décembre 2003 et novembre 2004 (899 en total) présentaient une augmentation linéaire nette tout au long des mois. La plupart des messages (68%) provenaient d’Espagne, le reste (32%) provenant d’Amérique Latine (Caraïbes, Amérique du Sud et Amérique Centrale) ; d’autre part, les usuaires étaient généralement des hommes (80%). La majorité des doutes (79%) concernaient l’infection VIH, le pourcentage touchant l’Hépatite étant nettement inférieur (17%). Le plus grand nombre de messages étaient enregistrés après les week-end (37% des questions les lundis et les mardis). Les soucis les plus fréquents de nos usuaires concernaient les facteurs de risque d’infection VIH (69%), suivis de la phase silencieuse (12,6%), les résultats des analyses (5,9%), les symptômes (4,7%), le diagnostique (2,7%) et le traitement (2,2%).

**Conclusion:** Nos résultats confirment la grande demande des services de “consultation avec un expert ” par Internet dans les champs du SIDA y de l’Hépatite. Le caractère anonyme, l’accès libre et la réponse immédiate sont des facteurs décisifs pour le succès de ce genre d’expérience.

**Conflit d’intérêts: Aucun. La Web est sponsorisée par la compagnie pharmaceutique Roche.**
